# Amygdalin Influences Bladder Cancer Cell Adhesion and Invasion *In Vitro*

**DOI:** 10.1371/journal.pone.0110244

**Published:** 2014-10-15

**Authors:** Jasmina Makarević, Jochen Rutz, Eva Juengel, Silke Kaulfuss, Igor Tsaur, Karen Nelson, Jesco Pfitzenmaier, Axel Haferkamp, Roman A. Blaheta

**Affiliations:** 1 Department of Urology, Goethe University Hospital, Frankfurt am Main, Germany; 2 Institute of Human Genetics, University Medical Center Göttingen, Göttingen, Germany; 3 Department of Vascular and Endovascular Surgery, Goethe University Hospital, Frankfurt am Main, Germany; 4 Department of Urology, Evangelical Hospital Bielefeld, Bielefeld, Germany; Innsbruck Medical University, Austria

## Abstract

The cyanogenic diglucoside amygdalin, derived from Rosaceae kernels, is employed by many patients as an alternative anti-cancer treatment. However, whether amygdalin indeed acts as an anti-tumor agent is not clear. Metastasis blocking properties of amygdalin on bladder cancer cell lines was, therefore, investigated. Amygdalin (10 mg/ml) was applied to UMUC-3, TCCSUP or RT112 bladder cancer cells for 24 h or for 2 weeks. Tumor cell adhesion to vascular endothelium or to immobilized collagen as well as tumor cell migration was examined. Effects of drug treatment on integrin α and β subtypes, on integrin-linked kinase (ILK) and total and activated focal adhesion kinase (FAK) were also determined. Integrin knock-down was carried out to evaluate integrin influence on migration and adhesion. A 24 h or 2 week amygdalin application distinctly reduced tumor cell adhesion and migration of UMUC-3 and RT112 cells. TCCSUP adhesion was also reduced, but migration was elevated under amygdalin. Integrin subtype expression was significantly and specifically altered by amygdalin depending on the cell line. ILK was moderately, and activated FAK strongly, lost in all tumor cell lines in the presence of amygdalin. Knock down of β1 integrin caused a significant decrease in both adhesion and migration of UMUC-3 cells, but a significant increase in TCCSUP adhesion. Knock down of β4 integrin caused a significant decrease in migration of RT112 cells. Since the different actions of amygdalin on the different cell lines was mirrored by β1 or β4 knock down, it is postulated that amygdalin influences adhesion and migratory properties of bladder cancer cells by modulating β1 or β4 integrin expression. The amygdalin induced increase in TCCSUP migratory behavior indicates that any anti-tumor benefits from amygdalin (seen with the other two cell lines) may depend upon the cancer cell type.

## Introduction

The use of complementary and alternative medicine (CAM) has steadily increased over the past decades. CAM includes non-conventional therapy such as homeopathy, vitamin therapy, phytomedicine and traditional Chinese medicine, acupuncture and yoga [1]. The consumption of natural products is most wide spread. Up to 80% of cancer patients in the United States [2], and more than 50% of cancer patients in Europe use CAM together with or in place of conventional therapy [3]. Dissatisfaction with conventional treatment and reduction of chemotherapeutic side effects are the most commonly given reasons for the use of CAM [4], [5].

In contrast to the wide spread use of natural compounds, information about their therapeutic effectivity is sparse. The discrepancy between use and factual benefit is particularly apparent with the cyanogenic diglucoside amygdalin (D-mandelonitrile-β-gentiobioside), present in the kernels of fruits from Rosaceae species such as Prunus persica (peach), Prunus armeniaca (apricot) and Prunus amygdalus amara (bitter almond). Amygdalin was first isolated in 1873. Since the 1920s, amygdalin has been orally applied to treat cancer patients in the United States. In the 1950s, an intravenous form of amygdalin was synthesized and patented as laetrile [6]. Although laetrile is chemically different from amygdalin, the terms are used interchangeably, making interpretation of clinical data difficult. The present report exclusively refers to “amygdalin”.

Amygdalin was one of the most popular, non-conventional, anti-cancer treatments in the 1970s and by 1978, 70,000 US cancer patients had used amygdalin [7]. Still, evidence based research on amygdalin was and is sparse and its benefit controversial. Proponents consider amygdalin a natural cancer cure, whereas opponents warn that amygdalin is ineffective and even toxic. Randomized clinical trials and follow-up studies have never been carried out. A clinical study sponsored by the National Cancer Institute 30 years ago did not reveal signs of tumor regression [8], whereas a retrospective analysis of 67 tumor patients taking amygdalin reported 2 complete and 4 partial responses [9]. Ambivalence has also been reflected in case reports, where amygdalin was ineffective in five and effective in four cases [6].

The present study was designed to evaluate whether amygdalin alters metastatic tumor cell progression in vitro since invasion and metastasis are critical steps in malignant tumor progression and the main cause of treatment failure. Therefore, interfering with the tumor cell invasion cascade might be an innovative option to counteract metastatic tumor dissemination. Employing a panel of bladder cancer cell lines, the efficacy of amygdalin to block tumor-matrix and tumor endothelial interaction was evaluated. Additionally, the capability of amygdalin to prevent motile spreading was assessed. A cohort of adhesion molecules is involved in the complex process of tumor cell dissemination. Since adhesion receptors of the integrin α and β family are closely involved in tumor cell binding and transendothelial penetration, these were the objects of investigation. The membranous integrin receptor expression profile, as well as the intracellular protein content of each subtype, was compared in amygdalin treated and non-treated cells. siRNA knock down studies were also carried out to investigate those parameters altered by amygdalin, which may have clinical relevance. The in vitro data presented here point to significant adhesion and invasion blocking effects of amygdalin, probably induced by altering β1 or β4 integrin expression.

## Materials and Methods

### Cell culture

RT112, UMUC-3 (ATCC/LGC Promochem GmbH, Wesel, Germany) and TCCSUP (DSMZ, Braunschweig, Germany) bladder carcinoma cells were grown and subcultured in RPMI 1640, 10% fetal calf serum (FCS), 20 mM HEPES-buffer, 1% glutamax and 1% penicillin/streptomycin (all: Gibco/Invitrogen; Karlsruhe, Germany). Subcultures from passages 7–24 were selected for experimental use. Human endothelial cells (HUVEC) were isolated from human umbilical veins and harvested by enzymatic treatment with dispase (Gibco/Invitrogen). HUVEC were grown in Medium 199 (M199; Biozol, Munich, Germany), supplemented with 10% FCS, 10% pooled human serum, 20 µg/ml endothelial cell growth factor (Boehringer, Mannheim, Germany), 0.1% heparin, 100 ng/ml gentamycin and 20 mM HEPES-buffer (pH 7.4). Subcultures from passages 2–6 were selected for experimental use. HUVEC were used in the study. The institutional ethics committee of the Goethe-University Hospital, Frankfurt, Germany, approved the investigation and waived the need for consent, since HUVEC were used anonymously for in vitro assays with no link to patient data.

### Amygdalin treatment

Amygdalin from apricot kernels (Sigma-Aldrich, Taufkirchen, Germany) was freshly dissolved in tumor cell culture medium and then added to tumor cells at a concentration of 10 mg/ml for either 24 h or for 2 weeks [10] to evaluate acute versus chronic treatment. Controls received tumor cell culture medium alone. In all experiments, treated tumor cell cultures were compared to the non-treated ones. To exclude toxic effects of amygdalin, cell viability was determined by trypan blue (Gibco/Invitrogen).

### Tumor cell adhesion

To analyze tumor cell adhesion, HUVEC were transferred to 6-well multiplates (Sarstedt, Nürnbrecht, Germany) in complete HUVEC-medium. When confluent, RT112, UMUC-3 or TCCSUP cells were detached from the culture flasks by accutase treatment (PAA Laboratories, Cölbe, Germany) and 0.5×10^6^ cells were then added to the HUVEC monolayer for 30, 60 or 120 min. Subsequently, non-adherent tumor cells were washed off using warmed (37°C) Medium 199. The remaining cells were fixed with 1% glutaraldehyde. Adherent tumor cells were counted in five different fields of a defined size (5×0.25 mm^2^) using a phase contrast microscope and the mean cellular adhesion rate was calculated.

### Attachment to immobilized collagen

6-well plates were coated with collagen G (extracted from calfskin, consisting of 90% collagen type I and 10% collagen type III; Biochrom, Berlin, Germany; diluted to 400 µg/ml in PBS) overnight. Plastic dishes served as the background control. Plates were washed with 1% BSA (bovine serum albumin) in PBS to block nonspecific cell adhesion. 0.5×10^6^ tumor cells were then added to each well and left for 60 min incubation. Subsequently, non-adherent tumor cells were washed off, the remaining adherent cells were fixed with 1% glutaraldehyde and counted microscopically. The mean cellular adhesion rate, defined by adherent cells_coated well_ − adherent cells_background_, was calculated from five different observation fields.

### Measurement of tumor cell migration

Serum induced chemotactic movement was examined using 6-well Transwell chambers (Greiner, Frickenhausen, Germany) with 8- µm pores. 0.5×10^6^ RT112, UMUC-3 or TCCSUP cells/ml were placed in the upper chamber in serum-free medium, either free of amygdalin (terminated “amygdalin A”) or containing amygdalin (terminated “amygdalin B”). Serum free medium in the upper chamber and 10% serum in the lower chamber provided the serum gradient necessary for tumor cell migration in this model. After 20 h incubation, the upper surface of the Transwell membrane was gently wiped with a cotton swab to remove cells, which had not migrated. Cells which had moved towards the serum gradient to the lower surface of the membrane were stained using hematoxylin and counted microscopically. The mean migration rate was calculated from five different observation fields.

### Integrin surface expression

Tumor cells were washed in blocking solution (PBS, 0.5% BSA) and then incubated for 60 min at 4 C with phycoerythrin (PE)-conjugated monoclonal antibodies directed against the following integrin subtypes: Anti-α1 (IgG1; clone SR84), anti-α2 (IgG2a; clone 12F1–H6), anti-α3 (IgG1; clone C3II.1), anti-α4 (IgG1; clone 9F10), anti-α5 (IgG1; clone IIA1), anti-α6 (IgG2a; clone GoH3), anti-β1 (IgG1; clone MAR4), anti-β3 (IgG1; clone VI-PL2) or anti-β4 (IgG2a; clone 439–9B; all: BD Pharmingen, Heidelberg, Germany). Integrin expression of tumor cells was then measured using a FACscan (BD Biosciences, Heidelberg; FL-2H (log) channel histogram analysis; 1×10^4^ cells/scan) and expressed as mean fluorescence units. A mouse IgG1-PE (MOPC-21) or IgG2a-PE (G155–178; all: BD Biosciences) was used as an isotype control.

### Western blotting

To investigate integrin content, tumor cell lysates were applied to a 7% polyacrylamide gel and electrophoresed for 90 min at 100 V. The protein was then transferred to nitrocellulose membranes. After blocking with non-fat dry milk for 1 h, the membranes were incubated overnight with the monoclonal antibodies listed above. Additionally, integrin-related signaling was explored by anti-integrin-linked kinase (ILK; clone 3, dilution 1∶1000), anti-focal adhesion kinase (FAK; clone 77, dilution 1∶1000) and anti-phospho-specific FAK (pY397; clone 18, dilution 1∶1000) antibodies (all: BD Biosciences). HRP-conjugated goat-anti-mouse IgG (Upstate Biotechnology, Lake Placid, NY, USA; dilution 1∶5.000) served as the secondary antibody. The membranes were briefly incubated with ECL detection reagent (ECL™, Amersham/GE Healthcare, München, Germany) to visualize the proteins and then analyzed by the Fusion FX7 system (Peqlab, Erlangen, Germany). β-actin (1∶1.000; Sigma, Taufenkirchen, Germany) served as the internal control.

Gimp 2.8 software was used to perform pixel density analysis of the protein bands. The ratio of protein intensity/β-actin intensity was calculated, and expressed in percentage, related to controls set to 100%.

### siRNA knock down studies

Tumor cells (3×10^5^/6-well) were transfected with small interfering RNA (siRNA) directed against integrin β1 (2 µM, target sequence: AAAAGTCTTGGAACAGATCTG, HS_ITGB1_5, Qiagen, Hilden, Germany) or integrin β4 (2 µM, target sequence: GTGGATGAGTTCCGGAATAAA; Hs_ITGB4_5, Qiagen) with a siRNA/transfection reagent (HiPerFect Transfection Reagent; Qiagen) ratio of 1∶6. Non-treated cells and cells treated with 5 nM control siRNA (All stars negative control siRNA; Qiagen) served as controls. Subsequently, tumor cell adhesion to immobilized collagen as well as tumor cell migration was analyzed as indicated above.

### Statistics

All experiments were performed 3–6 times. Statistical significance was determined by the Wilcoxon–Mann-Whitney-U-test. Differences were considered statistically significant at a p value less than 0.05.

## Results

### Amygdalin diminishes tumor-endothelium and tumor matrix interaction

**Figure 1:**
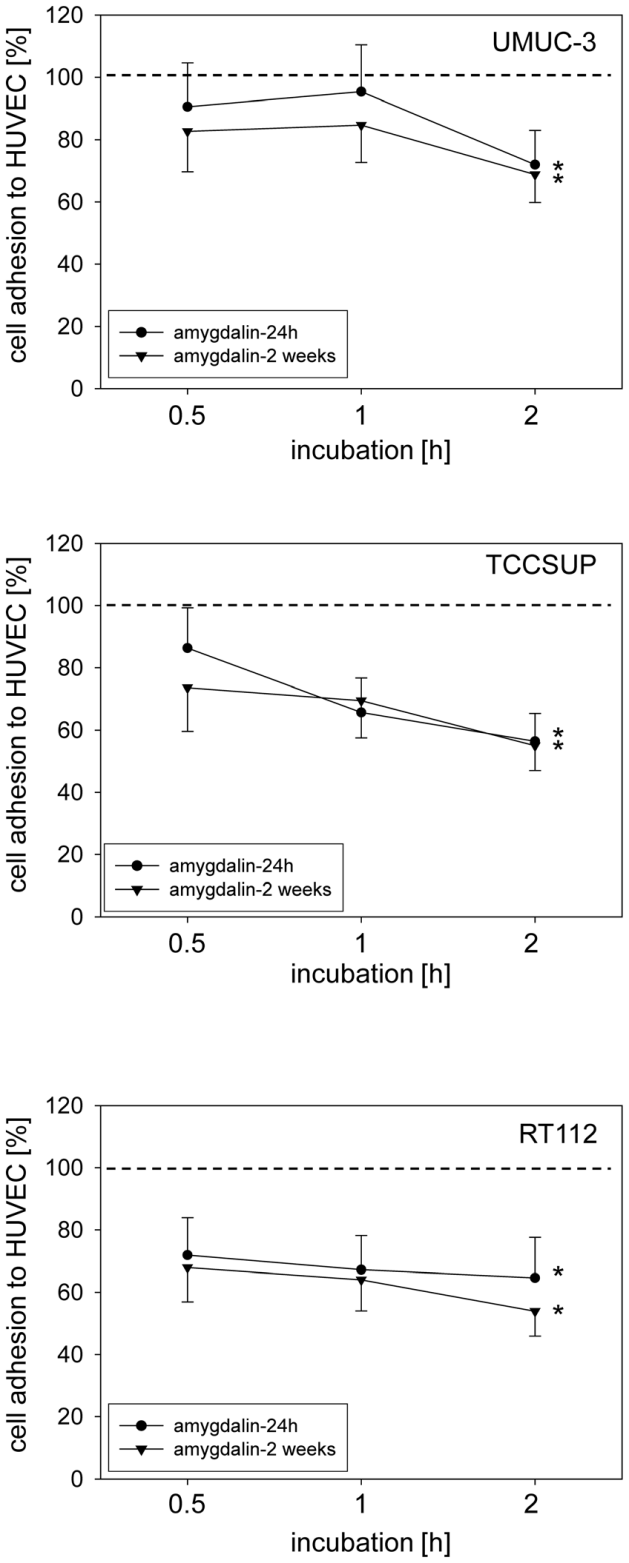
Adhesion of UMUC-3, TCCSUP and RT112 bladder cancer cells to HUVEC. Tumor cells were treated with 10 mg/ml amygdalin for either 24 h or for 2 weeks. Controls received cell culture medium alone. 0.5×10^6^ tumor cells/well were added to HUVEC monolayers for 0.5, 1 and 2 h. Mean adherent tumor cells from five fields was calculated and depicted as percentage of the 100% control (dotted line). One representative of six experiments is shown. *indicates significant difference to controls.

**Figure 2:**
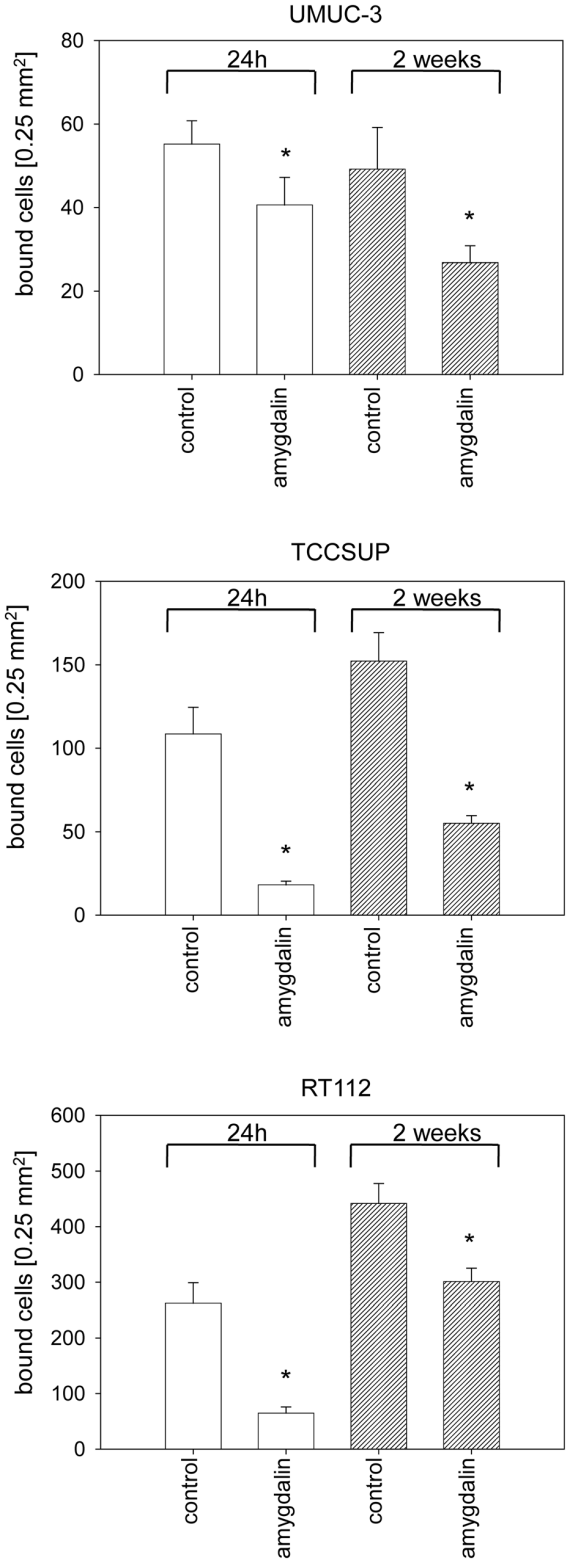
Adhesion of UMUC-3, TCCSUP and RT112 bladder cancer cells to immobilized collagen. Tumor cells were treated with 10 mg/ml amygdalin for either 24 h or for 2 weeks. Cells not treated with amygdalin served as the controls. 0.5×10^6^ cells/well were added to immobilized collagen for 60 min. Mean number of adherent tumor cells from five fields was calculated. One representative of six experiments is shown. *indicates significant difference to controls.

**A**mygdalin significantly reduced attachment of all three bladder cancer cell lines to HUVEC when compared to untreated cells (fig. 1). Cell adhesion of TCCSUP and RT112 was more strongly altered by amygdalin than that of UMUC-3 cells. No difference between short-term (24 h) and long-term (2 weeks) amygdalin treatment was apparent. The binding capacity of UMUC-3, TCCSUP and RT112 cells to immobilized collagen was also significantly down-regulated, compared to controls (fig. 2). Extending the treatment period from 24 h to 2 weeks did not further increase the blocking potential of amygdalin. No sign of toxicity due to amygdalin was detected by the trypan blue exclusion test.

### Amygdalin alters the migratory behavior of tumor cells

**Figure 3:**
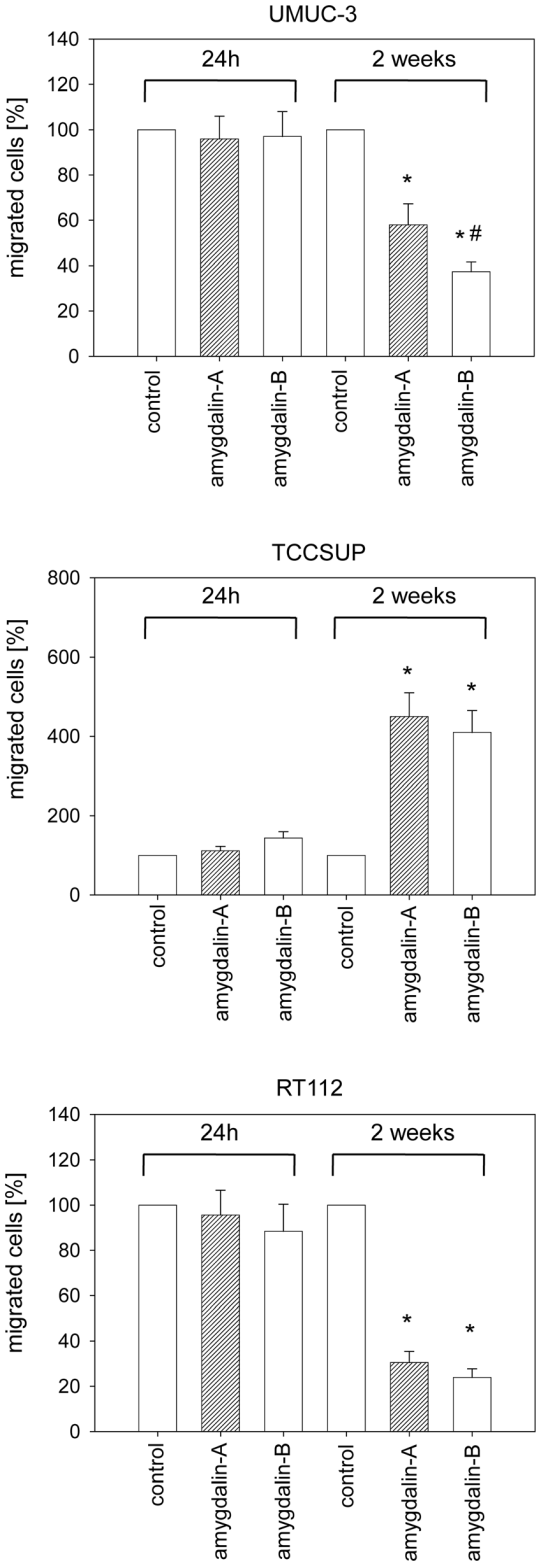
Effect of amygdalin on bladder cancer cell migration. Tumor cells treated with amygdalin for 24 h or for 2 weeks were seeded in the upper chamber with a chemo-attractant in the lower well. Cells were allowed to move for 20 h, either in amygdalin-free medium (amygdalin-A) or in amygdalin-containing medium (amygdalin-B). Cells migrating to the lower membrane surface were counted. Controls were set to 100%. One representative of six experiments is shown. *  =  significant difference to controls. #  =  significant difference between amygdalin-A and amygdalin-B.

Exposing the tumor cells to amygdalin for 24 h did not change their migratory activity (fig. 3). However, after a 2 week pretreatment period the number of UMUC-3 and RT112 cells beneath the Transwell chamber membrane was reduced, compared to the untreated control cells. The inhibitory effect in UMUC-3 cells was more pronounced when amygdalin remained in the cell culture medium during the 20 h migratory incubation (“amygdalin B”), compared to amygdalin free medium (“amygdalin A”). This difference in the 20 h migratory incubation with or without amygdalin was not detected in the RT112 cells, where migration was blocked to a similar extent. In contrast to RT112 and UMUC-3 cells, a massive increase in TCCSUP cell migration after 2 weeks amygdalin pretreatment was noted.

### Amygdalin acts on integrin α and β surface expression

**Figure 4:**
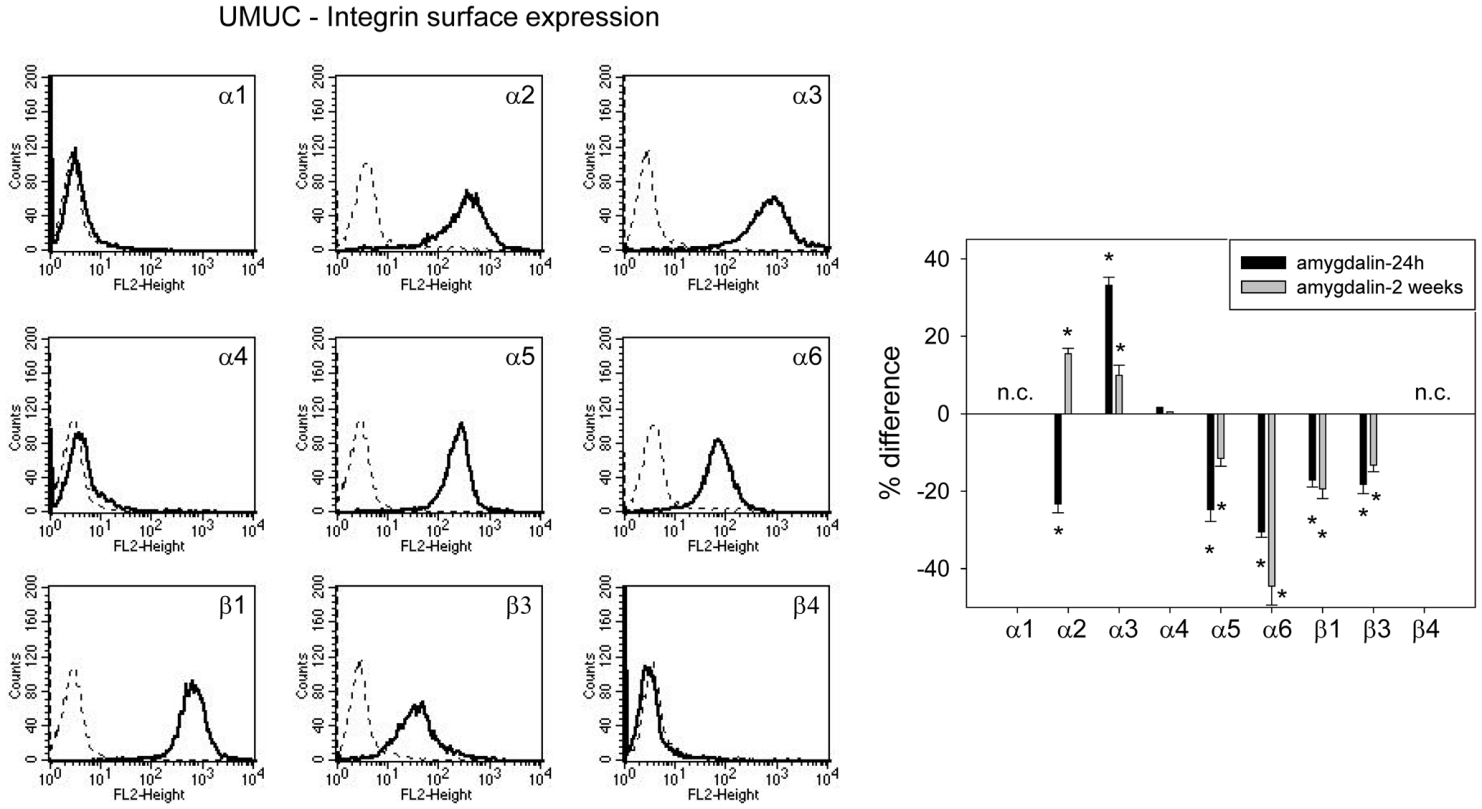
FACS analysis of integrin α and β subtype expression on UMUC-3 cells. The left panel depicts integrin expression as histogram plots with a dotted line indicating background fluorescence and a solid line indicating specific fluorescence in untreated cells. The right panel shows integrin subtype expression after 24 h and 2 weeks amygdalin exposure, compared to controls set at 100%. n.c.  =  not calculated. * indicates significant difference to controls.

**Figure 5:**
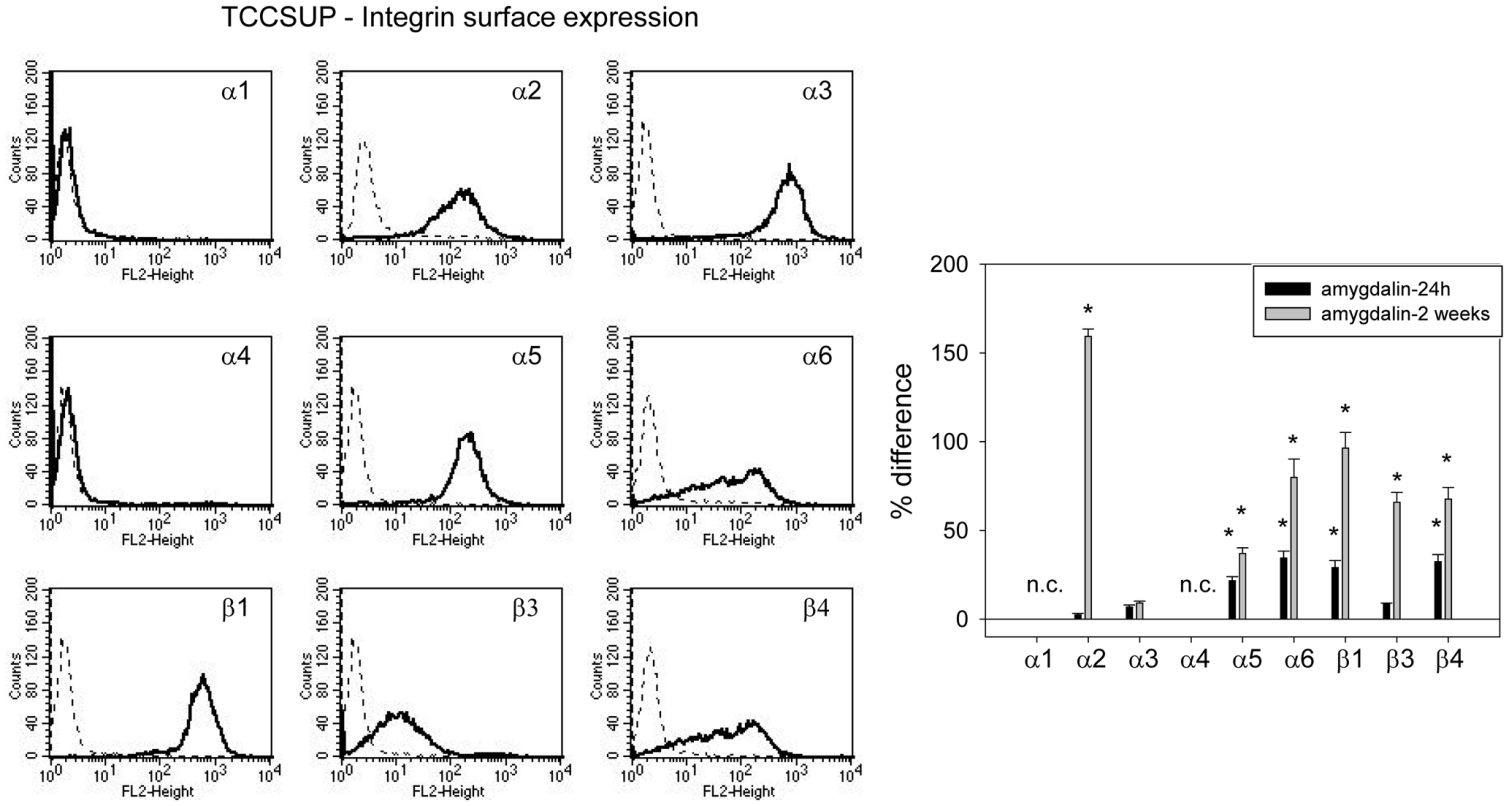
FACS analysis of integrin α and β subtype expression on TCCSUP cells. The left panel depicts integrin expression as histogram plots with a dotted line indicating background fluorescence and a solid line indicating specific fluorescence. The right panel shows integrin subtype expression after 24 h and 2 weeks amygdalin exposure, compared to controls set at 100%. n.c.  =  not calculated. * indicates significant difference to controls.

**Figure 6:**
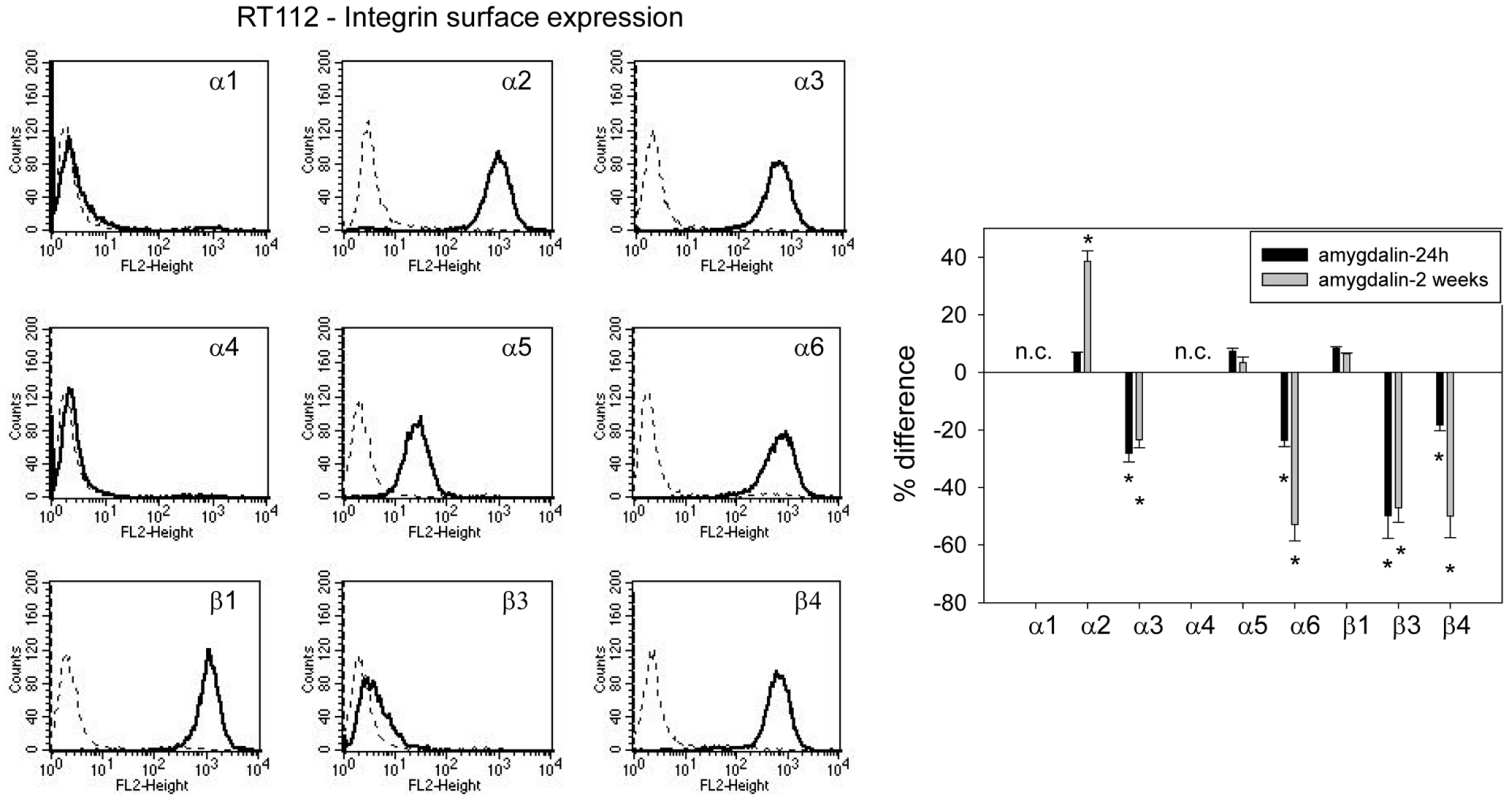
FACS analysis of integrin α and β subtype expression on RT112 cells. The left panel depicts integrin expression as histogram plots with a dotted line indicating background fluorescence and a solid line indicating specific fluorescence. The right panel shows integrin subtype expression after 24 h and 2 weeks amygdalin exposure, compared to controls set at 100%. n.c.  =  not calculated. * indicates significant difference to controls.

The integrin subtypes α2, α3, α5, α6, β1 and β3 were strongly expressed on UMUC-3 cells, α4 was very moderately expressed and α1 and β4 were not expressed (fig. 4, left). Amygdalin elevated α3 but reduced α5, α6, β1 and β3, independent from exposure time. No amygdalin induced modification was noted in α4 integrin. The α2 receptor was down-regulated after 24 h but up-regulated after 2 weeks amygdalin exposure (fig. 4, right). TCCSUP cells distinctly expressed the α2, α3, α5, α6, β1 and β4 integrin members (fig. 5, left). The β3 type was moderately present on the cell surface. Both α1 and α4 subtypes were not detectable. Amygdalin led to a significant increase in integrin α5, α6, β1 and β4 on TCCSUP, whereby the 2 weeks application induced stronger effects than the 24 h incubation (fig. 5, right). The α2 and β3 subtypes were enhanced after 2 weeks, but not after 24 h. α3 was not altered by amygdalin. RT112 cells were characterized by a high α2, α3, α6, β1 and β3 expression level (fig. 6, left). Integrin α5 was moderately expressed, and β3 was only slightly elevated over background. Integrins α1 and α4 were not expressed on RT112 cells. The integrins α3, α6, β3 and β4 were all suppressed by amygdalin (fig. 6, right). The effects on α3 and β3 did not depend on whether amygdalin had been applied for 24 h or 2 weeks, whereas α6 and β4 were diminished to a greater extent after 2 weeks, compared to 24 h. α2 distinctly increased after 2 weeks but not after 24 h, and α5 and β1 remained unchanged by amygdalin.

### Modifications of integrin proteins by amygdalin

**Figure 7:**
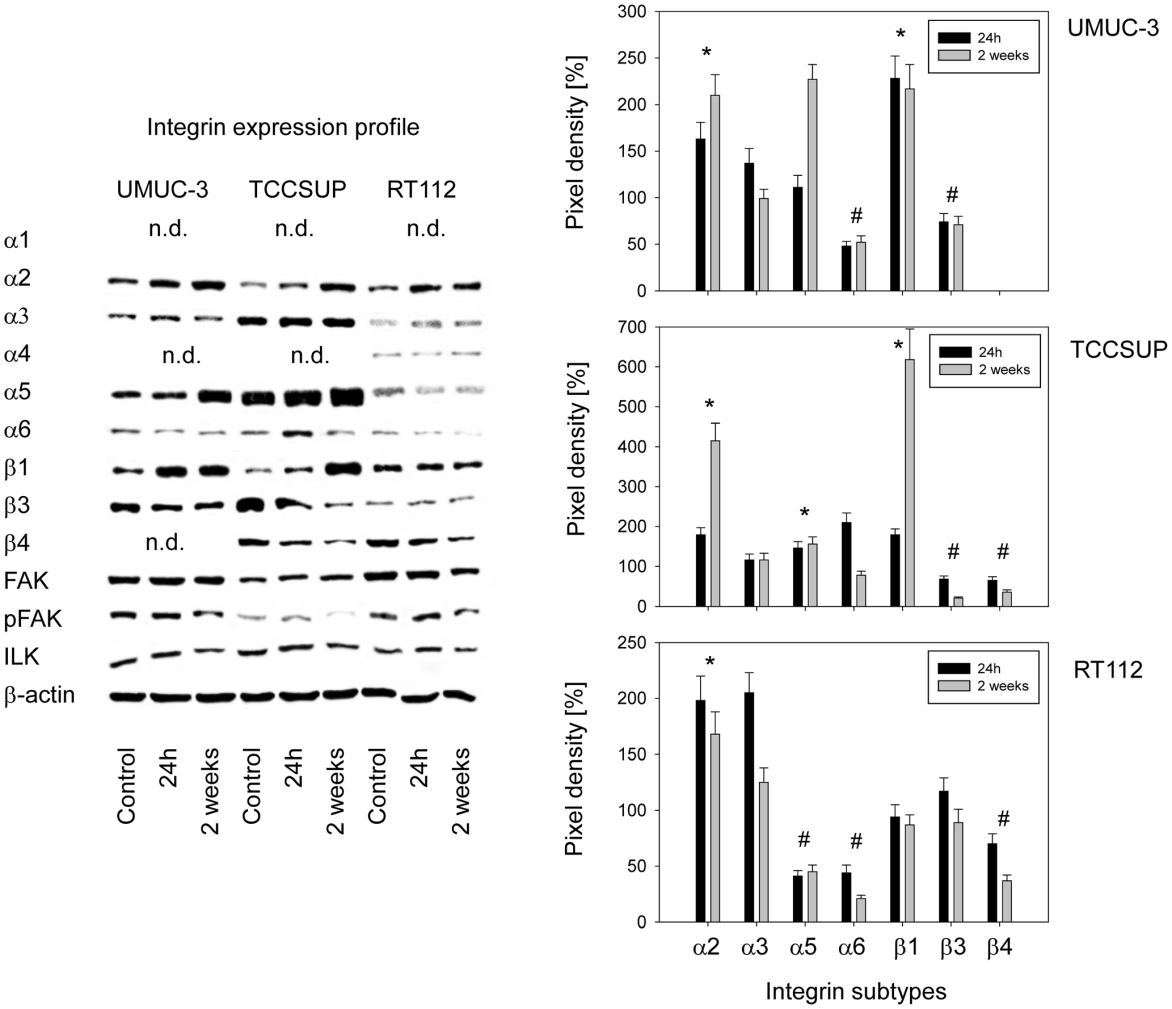
Intracellular integrin protein content of UMUC-3, TCCSUP and RT112 bladder cancer cells exposed to amygdalin for 24 h or 2 weeks. Controls remained untreated. β-actin served as the internal control. The figure shows one representative from three separate experiments. n.d. indicates “not detectable”. Quantification of integrin subtype expression is depicted on the right. Pixel density is given in percentage compared to controls not treated with amygdalin. *indicates significant increase after both 24 h and 2 weeks amygdalin treatment, #indicates significant decrease after both 24 h and 2 weeks amygdalin treatment, compared to controls.

Alterations of the integrin protein content induced by amygdalin are shown in figure 7 (left) and quantification is expressed as percentage difference between control tumor cells and tumor cells treated with amygdalin (right). In UMUC-3 cells, α6 and β3 were suppressed by both 24 h and 2 weeks amygdalin application, whereas α2 and β1 were up-regulated. An amygdalin induced increase in α5 was apparent but this effect was restricted to the 2 week amygdalin application. α3 integrin was slightly enhanced over controls after 24 h but not after 2 weeks. The integrins α1, α4 and β4 were not detectable by western blotting. The integrin related signaling proteins ILK and pFAK were diminished after 2 weeks amygdalin application. Similar action was exerted on TCCSUP cells, since α2, α5 and β1 increased and β3 decreased under amygdalin (2 weeks >24 h). In contrast to UMUC-3, α6 was enhanced after 24 h but reduced after 2 weeks. β4, not expressed in UMUC-3, was down-regulated by amygdalin (2 weeks >24 h). The 2 week amygdalin application led to a loss of pFAK and slightly diminished ILK. Evaluation of RT112 revealed increased α2 integrin caused by 24 h or 2 week amygdalin treatment. α6 and β4 integrins were down-regulated with 2 week >24 h. There was also a slight increase of α3 after 24 h but not after 2 weeks, a phenomenon also seen in UMUC-3 cells. Opposed to UMUC-3 and TCCSUP, the α5 subtype in RT112 was only moderately detectable in the control cells and was further diminished following drug treatment. Amygdalin additionally influenced integrin related signaling in RT112, evidenced by diminished FAK and pFAK.

### β1 and β4 integrin knockdown

**Figure 8:**
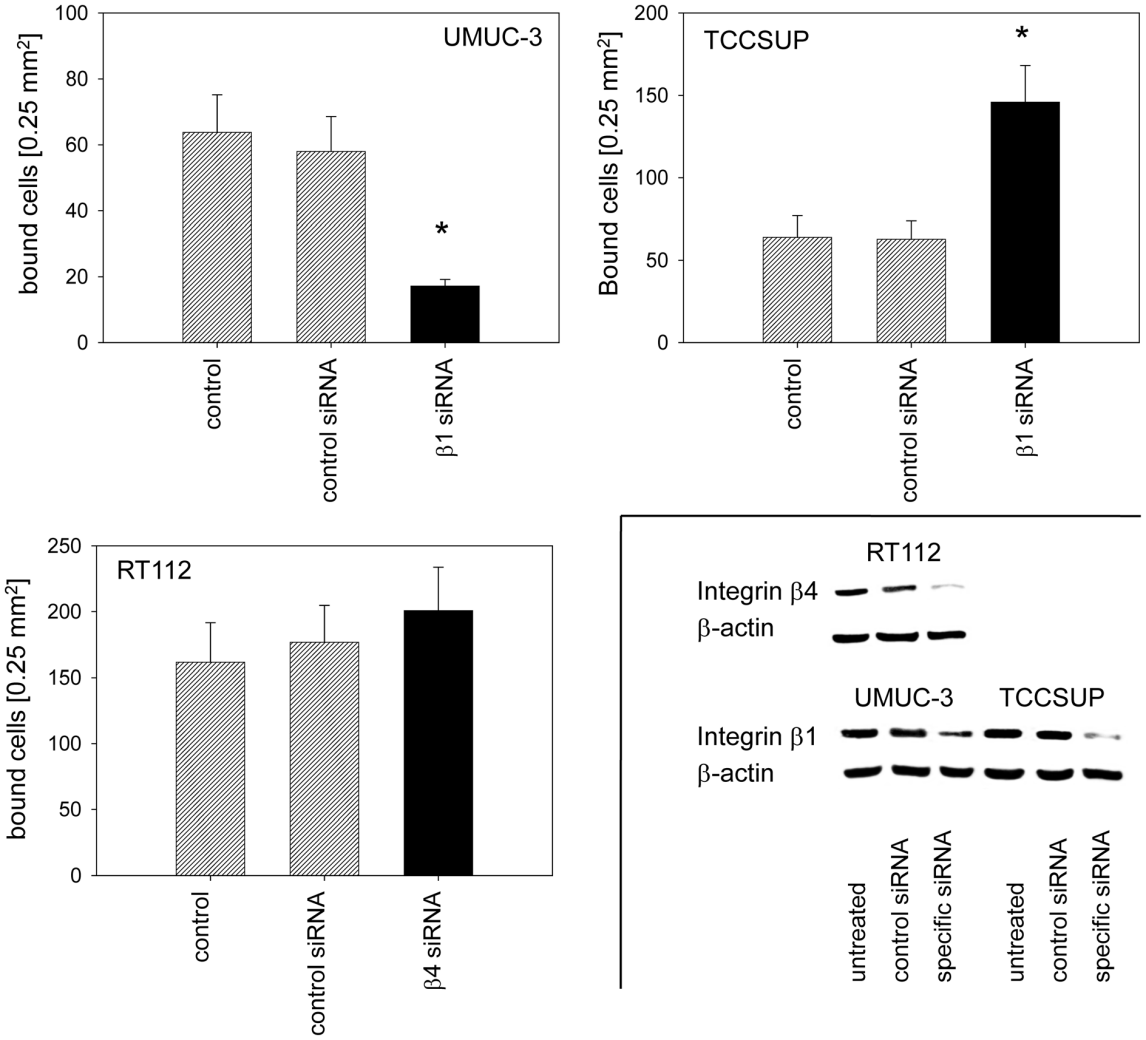
Influence of β1 (UMUC-3, TCCSUP) or β4 (RT112) knock-down on bladder cancer cell adhesion to collagen. Tumor cells were transfected with integrin β1 or β4 siRNA. Non- treated cells (control) and cells treated with scrambled siRNA (siRNA control) served as controls. Efficacy of receptor knockdown was evaluated by western blotting (lower right). One representative of six experiments is shown. *indicates significant difference to the controls.

**Figure 9:**
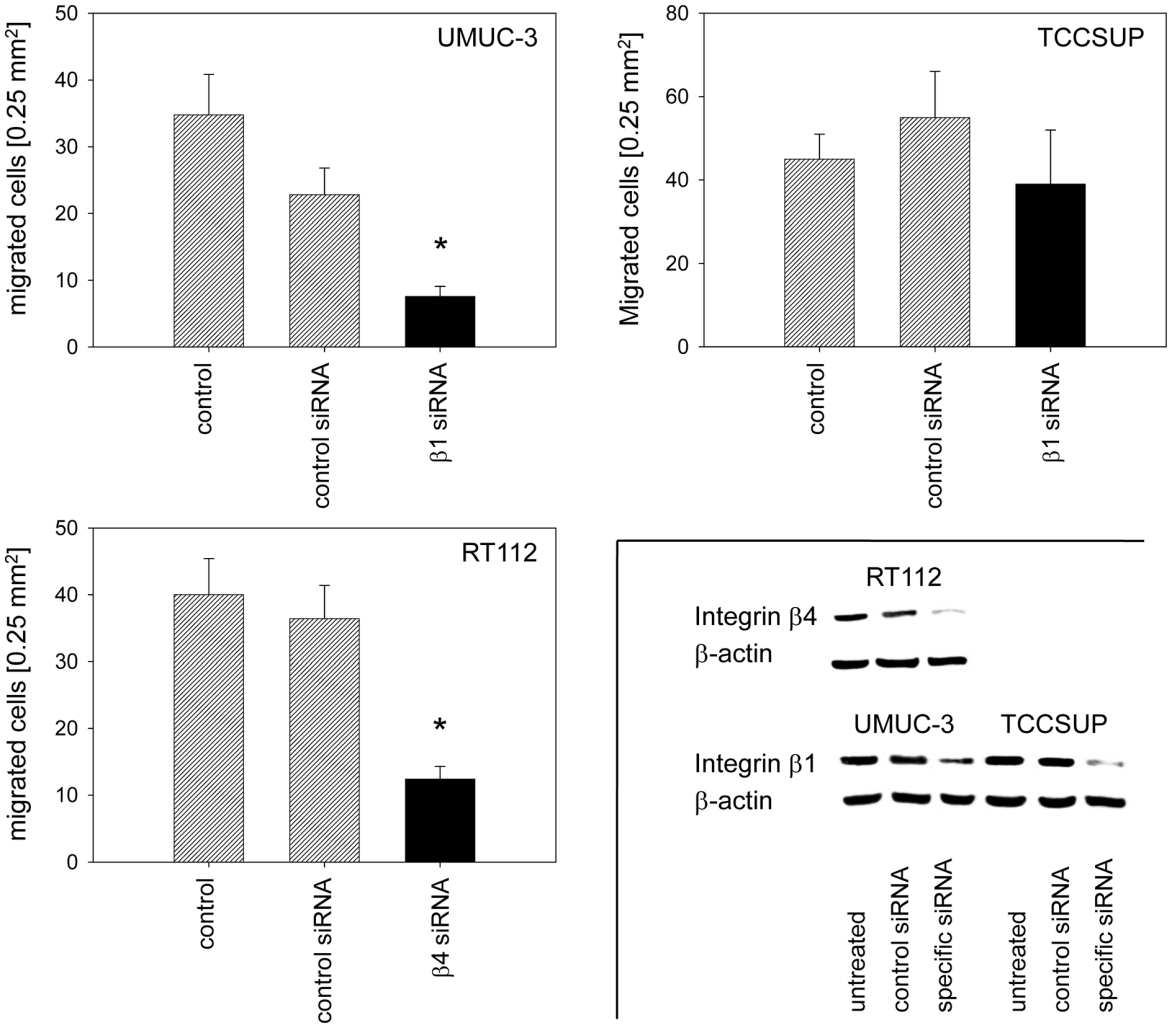
Knock-down of β1 (UMUC-3, TCCSUP) or β4 (RT112) integrin alters chemotaxis. Tumor cells were transfected with integrin β1 or β4 siRNA or scrambled siRNA (siRNA control). Controls remained untreated (control). Values are shown as migrated cells per 0.25 mm^2^. One representative of six experiments is shown. * indicates significant difference to the untreated control. Insert taken from figure 8.

Amygdalin distinctly altered the integrin expression profile of all three bladder cancer cell lines. To investigate whether integrin modifications are relevant to metastatic progression, knock down studies were carried out with the β-integrin members serving as representatives. Since β1 (but not β3 and β4) was highly expressed on control UMUC-3 and TCCSUP and significantly diminished by amygdalin, β1 was knocked down in these cells and adhesion and migration experiments repeated (fig. 8). The β1 integrin was also highly expressed on RT112 but not modified by amygdalin, in contrast to β4, which fulfilled both criteria, i.e. high initial expression and significant modulation by amygdalin. Therefore, β4 was knocked-down in the RT112 cell line before subjection to the adhesion and migration assay (fig. 8, lower right). Loss of β1 was accompanied by a significant reduction in UMUC-3 binding (fig. 8) and migration (fig. 9). On the other hand, TCCSUP binding to collagen was enhanced (fig. 8), whereas TCCSUP migration was not influenced by β1 knock down (fig. 9). Down-regulating the β4 integrin in RT112 cells did not alter the adhesion properties (fig. 8) but massively blocked migration (fig. 9).

## Discussion

Since tumor cell interaction with the vascular endothelium is necessary for tumor cells to leave the blood stream for establishment at secondary sites, interfering with this process is crucial to hindering metastasis. The present report shows that amygdalin significantly inhibits attachment of bladder cancer cells to endothelial cells. Since amygdalin reduces the number of tumor cells, fewer cells may crawl beneath the endothelial layer to establish contact with matrix proteins to metastasize. The next step in metastasis involves the collagen matrix. Interaction of tumor cells with collagen must not only occur to escape the primary tumor but also to permit invasive spreading into the target organ, once the tumor cells have traversed the endothelial blood barrier [11]. In the presence of amygdalin, the tumor cells used in this investigation lost their capacity to bind to immobilized collagen. Therefore, amygdalin may slow metastatic progression by preventing mechanical contacts of circulating tumor cells to the vessel wall and the sub-endothelial matrix. Tumor dissemination, however, is not restricted to binding. The cells must also detach from matrix proteins to invade tissue. Amygdalin influenced the migration capacity of all the bladder cancer cell lines after 2 weeks, but not after 24 h, indicating that long-term treatment may be necessary to alter tumor cell motility.

Amygdalin's action on the different tumor cell lines was not identical. Migration of UMUC-3 and RT112 was blocked, whereas the number of migrating TCCSUP cells increased with amygdalin. This means that although amygdalin diminishes the attachment rate in all cancer cell lines, there is a risk that chronic amygdalin exposure to a few remaining cells of a particular tumor subtype like TCCSUP may result in increased locomotive activity. Whether due to an acquired or intrinsic resistance or due to the development of undesired feedback loops remains unclear, but this does indicate that not all bladder cancer patients may profit equally well from amygdalin. In line with this speculation, it has recently been shown that resistance development is accompanied by a functional switch of integrin receptors, driving tumor cells to high motility [12].

Chen et al. recently speculated that tumor cells with a high migration speed are much more likely to metastasize than those with a low migration speed [13]. This, however, could not be confirmed by another investigation in which the number of migrating tumor cells and the distance and speed of migration did not correlate with the malignant potential of the tumor cells [14]. Rather, the direction of cell motility indicated the malignant potential of the cancer cells [14]. To gain further understanding, animal studies have just been initiated to explore in vivo tumor progression under amygdalin treatment.

The role of integrins in forming cell-cell and cell-matrix bonds necessary for adhesion, extravasation and migration has been broached [15], [16], whereby the β1 integrin subunit was shown to play a central role in the bladder cancer cell line T24. However, not all bladder cancer cell lines are characterized by the same integrin pattern. In the present investigation amygdalin modified adhesion and migration and altered integrin expression profiles differently in different cell lines. Integrin β4 was not expressed on UMUC-3 but on RT112 and TCCSUP, whereas β3 was only marginally detectable on RT112 but strongly detectable in UMUC-3 and TCCSUP cells. An investigation involving the influence of valproic acid on bladder cell adhesion to collagen [17], has also revealed modification of the integrin expression profile depending on the cell line employed. Other investigators have reported differing integrin subfamilies in UMUC, T24, J82, RT-4, 253J and Hu456 cells [18], [19]. Each cell line may, therefore, possess a characteristic receptor set and drug treatment may influence integrin subfamilies differently.

Investigations on prostate cancer cells have revealed that the initial integrin profile of a particular tumor clone may determine its molecular response to drug treatment [20]. Indeed, amygdalin influenced the integrin composition of the evaluated bladder tumor cells differently. In the UMUC-3 cells β1 and β3 surface expression was diminished by amygdalin, but enhanced in TCCSUP cells. In RT112 cells β4 instead of β1 was altered by amygdalin. Intracellular and membrane integrin levels were also differently affected by amygdalin in UMUC-3 and TCCSUP cells. In UMUC-3 cells intracellular β1 integrin was elevated and surface expression was diminished, indicating that amygdalin induces translocation of β1 integrin away from the surface membrane. In TCCSUP cells β1 integrin expression was increased both intracellularly and on the surface membrane. Amygdalin induced a decrease in intracellular β3 integrin in both UMUC-3 and TCCSUP cells. Surface expression, however, was not the same, with UMUC-3 cells showing an amygdalin induced decrease in β3 integrin and TCCSUP cells showing an increase. The main translocation in TCCSUP cells from the cytoplasm to the surface membrane seems, therefore, to be directed at β3 integrin.

Removal of certain integrin subtypes from the cell surface is not the only mechanism regulating cancer cell adhesion. Integrin trafficking between intracellular compartments and the cell surface has been shown to be a necessary prerequisite to receptor removal at the base of cell protrusions. Recycling integrin back to the leading cell edge supports adhesion [21]. A preclinical study on renal cancer cells has demonstrated that both integrin receptor up- and down-regulation can drive cancer cells towards malignancy. Consequently, therapeutic intervention aimed at ‘re-translocating’ certain integrin molecules might provide an option to prevent malignancy [22].

The relevance of integrins for adhesion and migration was demonstrated by knock-down studies on β integrins with either high initial surface expression or strong amygdalin induced alteration. These criteria were fulfilled for β1 in UMUC-3 and TCCSUP cells and for β4 in RT112 cells. Suppression of β1 correlated well with reduced binding and migration activity of UMUC-3, which accords to in vitro studies with T24 and 5637 cells [23], [24]. Therefore, loss of β1 might be a mechanism whereby amygdalin slows UMUC-3 tumor dissemination. TCCSUP cells, however, behaved differently under β1 blockade. Binding events to collagen actually increased, indicating that this receptor in these cells blocks cell-cell or cell-matrix contacts. Differential integrin guided adhesive behavior of different tumor sublines has been previously observed. Blocking the α3 subunit has been shown to inhibit HCV29 bladder cancer cell attachment to the matrix proteins laminin and fibronectin but has an opposite effect on T24 and Hu456 cell adhesion. Similarly, blocking the α5 integrin inhibited HCV29 and BC3726 cell-matrix interaction, whereas binding of the bladder cancer cell lines T24 and Hu456 was enhanced [25].

Since migration in TCCSUP is not modified by β1 integrin, this receptor cannot account for the increased locomotive activity of these cells in the presence of amygdalin. It is possible that α2 and/or β3 integrin may be responsible for their increased motility since both were distinctly elevated when TCCSUP cells were treated with amygdalin for 2 weeks, but not for 24 h, matching the motile behavior of TCCSUP becoming elevated after 2 weeks but not after 24 h.

In RT112 cells a different action of amygdalin may be assumed. β4 integrin was shown to modulate migration but not adhesion, whereby loss of β4 significantly correlated with reduced chemotaxis. Presumably, amygdalin weakens RT112 migratory capacity by diminishing β4 expression.

Aside from the cell-type dependent action on the integrin receptors, amygdalin homogeneously diminished expression of ILK (minor) and activation of FAK (major) in all bladder tumor cell lines used in this investigation. In vitro and in vivo, as well as clinical studies, have implicated ILK over-expression in invasive bladder cancer playing an important role in epithelial-to-mesenchymal transition [26], [27]. Likewise, the integrin related protein FAK is critical for promoting urothelial cancer cell motility [28], with a relationship between cell adhesion force and FAK activation level [29]. Pharmacologic suppression of FAK has been shown to prevent cell migration in vitro [30] and inhibit metastasis in a murine bladder cancer model [31]. Down-regulating ILK and FAK by amygdalin might therefore be a pivotal step in counteracting tumor dissemination.

In conclusion, amygdalin's mode of action varies, depending on the initial tumor cell's integrin composition. Accumulation or loss of specific integrin members as well as integrin translocation and turnover may contribute to a less aggressive tumor phenotype in the presence of amygdalin. However, conclusions drawn from in vitro investigations cannot be carried over into the clinical setting. Animal studies are underway to evaluate the potential of amygdalin in vivo. The migratory behavior of amygdalin treated TCCSUP cells in vivo will receive particular attention, since amygdalin induced in vitro migration.

## References

[1] Fischer FH, Lewith G, Witt CM, Linde K, von Ammon K, et al (2014) High prevalence but limited evidence in complementary and alternative medicine: guidelines for future research. BMC Complement Altern Med 14: 46. 24499316 10.1186/1472-6882-14-46 PMC3931324

[2] Saghatchian M, Bihan C, Chenailler C, Mazouni C, Dauchy S, et al. (2014) Exploring frontiers: Use of complementary and alternative medicine among patients with early-stage breast cancer. Breast. In press. 10.1016/j.breast.2014.01.009 24529905

[3] Huebner J, Micke O, Muecke R, Buentzel J, Prott FJ, et al (2014) User Rate of Complementary and Alternative Medicine (CAM) of Patients Visiting a Counseling Facility for CAM of a German Comprehensive Cancer Center. Anticancer Res 34: 943–948. 24511037

[4] Gillett J, Ientile C, Hiscock J, Plank A, Martin JM (2012) Complementary and alternative medicine use in radiotherapy: what are patients using? J Altern Complement Med 18: 1014–1020. 22906192 10.1089/acm.2011.0334

[5] Citrin DL, Bloom DL, Grutsch JF, Mortensen SJ, Lis CG (2012) Beliefs and perceptions of women with newly diagnosed breast cancer who refused conventional treatment in favor of alternative therapies. Oncologist 17: 607–612. 22531358 10.1634/theoncologist.2011-0468 PMC3360900

[6] Milazzo S, Lejeune S, Ernst E (2007) Laetrile for cancer: a systematic review of the clinical evidence. Support Care Cancer 15: 583–595. 17106659 10.1007/s00520-006-0168-9

[7] Moss RW (2005) Patient perspectives: Tijuana cancer clinics in the post-NAFTA era. Integr Cancer Ther 4: 65–86. 15695477 10.1177/1534735404273918

[8] Moertel CG, Fleming TR, Rubin J, Kvols LK, Sarna G, et al (1982) A clinical trial of amygdalin (Laetrile) in the treatment of human cancer. N Engl J Med 306: 201–206. 7033783 10.1056/NEJM198201283060403

[9] Newell GR, Ellison NM (1980) Ethics and designs: laetrile trials as an example. Cancer Treat Rep 64: 363–365. 6996807

[10] Makarević J, Rutz J, Juengel E, Kaulfuss S, Reiter M, et al (2014) Amygdalin blocks bladder cancer cell growth in vitro by diminishing cyclin A and cdk2. Plos One 9: e105590. 25136960 10.1371/journal.pone.0105590 PMC4138189

[11] Geraldo S, Simon A, Elkhatib N, Louvard D, Fetler L, et al (2012) Do cancer cells have distinct adhesions in 3D collagen matrices and in vivo? Eur J Cell Biol 91: 930–937. 22939225 10.1016/j.ejcb.2012.07.005

[12] Juengel E, Makarević J, Reiter M, Mani J, Tsaur I, et al (2014) Resistance to the mTOR inhibitor temsirolimus alters adhesion and migration behavior of renal cell carcinoma cells through an integrin α5- and integrin β3-dependent mechanism. Neoplasia 16: 291–300. 24862756 10.1016/j.neo.2014.03.011 PMC4094828

[13] Chen J, Sprouffske K, Huang Q, Maley CC (2011) Solving the puzzle of metastasis: the evolution of cell migration in neoplasms. PLoS One 6: e17933. 21556134 10.1371/journal.pone.0017933 PMC3083389

[14] Weiger MC, Vedham V, Stuelten CH, Shou K, Herrera M, et al (2013) Real-time motion analysis reveals cell directionality as an indicator of breast cancer progression. PLoS One 8: e58859. 23527039 10.1371/journal.pone.0058859 PMC3602596

[15] Mierke CT, Frey B, Fellner M, Herrmann M, Fabry B (2011) Integrin α5β1 facilitates cancer cell invasion through enhanced contractile forces. J Cell Sci 124: 369–383. 21224397 10.1242/jcs.071985 PMC3021998

[16] Heyder C, Gloria-Maercker E, Hatzmann W, Niggemann B, Zänker KS, et al (2005) Role of the beta1-integrin subunit in the adhesion, extravasation and migration of T24 human bladder carcinoma cells. Clin Exp Metastasis 22: 99–106. 16086230 10.1007/s10585-005-4335-z

[17] Juengel E, Meyer dos Santos S, Schneider T, Makarevic J, Hudak L, et al (2013) HDAC inhibition suppresses bladder cancer cell adhesion to collagen under flow conditions. Exp Biol Med (Maywood) 238: 1297–1304. 24006305 10.1177/1535370213498975

[18] Liebert M, Washington R, Stein J, Wedemeyer G, Grossman HB (1994) Expression of the VLA beta 1 integrin family in bladder cancer. Am J Pathol 144: 1016–1022. 8178925 PMC1887346

[19] Laidler P, Gil D, Pituch-Noworolska A, Ciołczyk D, Ksiazek D, et al (2000) Expression of beta1-integrins and N-cadherin in bladder cancer and melanoma cell lines. Acta Biochim Pol 47: 1159–1170. 11996105

[20] Wedel S, Hudak L, Seibel JM, Makarević J, Juengel E, et al (2011) Impact of combined HDAC and mTOR inhibition on adhesion, migration and invasion of prostate cancer cells. Clin Exp Metastasis 28: 479–491. 21452015 10.1007/s10585-011-9386-8

[21] Jacquemet G, Green DM, Bridgewater RE, von Kriegsheim A, Humphries MJ, et al (2013) RCP-driven α5β1 recycling suppresses Rac and promotes RhoA activity via the RacGAP1-IQGAP1 complex. J Cell Biol 202: 917–935. 24019536 10.1083/jcb.201302041 PMC3776348

[22] Oertl A, Relja B, Makarevic J, Weich E, Höfler S, et al (2006) Altered expression of beta1 integrins in renal carcinoma cell lines exposed to the differentiation inducer valproic acid. Int J Mol Med 18: 347–354. 16820945

[23] Yamasaki M, Iwase M, Kawano K, Sakakibara Y, Suiko M, et al (2014) α-Lipoic acid suppresses migration and invasion via downregulation of cell surface β1-integrin expression in bladder cancer cells. J Clin Biochem Nutr 54: 18–25. 24426186 10.3164/jcbn.13-57 PMC3882485

[24] Chakraborty A, White SM, Guha S (2006) Granulocyte colony-stimulating receptor promotes beta1-integrin-mediated adhesion and invasion of bladder cancer cells. Urology 68: 208–213. 16844458 10.1016/j.urology.2006.01.046

[25] Lityńska A, Przybyło M, Pocheć E, Laidler P (2002) Adhesion properties of human bladder cell lines with extracellular matrix components: the role of integrins and glycosylation. Acta Biochim Pol 49: 643–650. 12422234

[26] Matsui Y, Assi K, Ogawa O, Raven PA, Dedhar S, et al (2012) The importance of integrin-linked kinase in the regulation of bladder cancer invasion. Int J Cancer 130: 521–531. 21351095 10.1002/ijc.26008

[27] Xiong D, Liou Y, Shu J, Li D, Zhang L, et al. (2014) Down-regulating ribonuclease inhibitor enhances metastasis of bladder cancer cells through regulating epithelial-mesenchymal transition and ILK signaling pathway. Exp Mol Pathol. In press. 10.1016/j.yexmp.2014.04.012 24768914

[28] Genua M, Xu SQ, Buraschi S, Peiper SC, Gomella LG, et al (2012) Proline-rich tyrosine kinase 2 (Pyk2) regulates IGF-I-induced cell motility and invasion of urothelial carcinoma cells. PLoS One 7: e40148. 22859931 10.1371/journal.pone.0040148 PMC3408023

[29] Ay C, Yeh CC, Hsu MC, Hurng HY, Kwok PC, et al (2012) Evaluation of the correlation between focal adhesion kinase phosphorylation and cell adhesion force using "DEP" technology. Sensors (Basel) 12: 5951–5965. 22778624 10.3390/s120505951 PMC3386723

[30] Lai KC, Hsu SC, Yang JS, Kuo CL, Ip SW, et al (2011) 2-(3-Methoxyphenyl)-6, 7-methylenedioxoquinolin-4-one, a novel synthetic compound, inhibited migration and invasion in TSGH8301 human bladder cancer cells. Hum Exp Toxicol 30: 1045–1052. 20930028 10.1177/0960327110386257

[31] Green TP, Fennell M, Whittaker R, Curwen J, Jacobs V, et al (2009) Preclinical anticancer activity of the potent, oral Src inhibitor AZD0530. Mol Oncol 3: 248–261. 19393585 10.1016/j.molonc.2009.01.002 PMC5527863

